# The stridulatory organ in Opetiidae and Phoridae (Diptera) and phylogenetic implications for the evolution of higher flies

**DOI:** 10.1038/s41598-023-48669-2

**Published:** 2023-12-04

**Authors:** Brian V. Brown, Dalton de Souza Amorim

**Affiliations:** 1 Department of Entomology, Natural History Museum of Los Angeles County, Los Angeles, USA; 2 Departmento de Biologia, Universidade de São Paulo, Ribeirão Preto, Brazil

**Keywords:** Taxonomy, Phylogenetics

## Abstract

Stridulatory sound-making organs evolved in a group of flies—the family Phoridae—by modifications of the microstructure of foreleg segments present in the shared ancestor of the clade (Phoridae + Opetiidae). The opetiids are the only group amongst the lower Cyclorrhapha in which plausible homologous structures could be found, though in a less derived condition. On the forefemur of *Opetia* there are numerous elongate, flattened microtrichia that in basal phorids are organized into a curved linear group (the scraper) which are scraped against a curved, ridged carina on the forecoxa (the file). The file was possibly derived from an extremely unusual set of three setae that have transverse sculpturing and sockets that limit lateral motion, and which are distributed across the opetiid forecoxa. In some phorid lineages, these setae seem to be fused into the forecoxa forming the linear ridged surface against which the scraper on the forefemur could be moved. The relationship between opetiids and phorids dates back to the Cretaceous, and this pattern of file and scraper can be clearly seen in some 100 mya Myanmar amber phorid fly fossils. These structures shared between opetiids and phorids suggest that these two families may be sister groups amongst the Platypezoidea. Different modifications of the forelegs of other higher flies may have similar roles.

## Introduction

Communication between individuals of a species has been a fundamental need since the origin of life. This communication evolved through various modalities during biological evolution—beginning with chemical communication at early stages of life in the ocean. In the terrestrial environment, most insects have chemical communication, while sound production is more limited, depending on the availability of organs to produce and detect sound, distance needed for sound to travel, and the avoidance of non-target and potentially hostile eavesdroppers. Producing sounds for the sake of territory protection, mating, warning, etc. appeared in different groups of insects and new structures for sound production are still being discovered, especially at the smaller scale at which insects live.

The family Phoridae, commonly known as humpbacked flies or scuttle flies, are among the largest groups of flies in the world^1,2^. The 4500 species now formally described are believed to represent less than 1% of the actual number of existing species. The range of structures and lifestyles of these tiny (0.4–6.0 mm long) flies are unmatched, at least within the Diptera, or true flies. Their species-richness, especially in tropical forests, is matched by few other groups.

The relationships of the Phoridae to other lower Cyclorrhapha, especially within the Platypezoidea, (comprised by Ironomyiidae, Lonchopteridae, Opetiidae, Platypezidae, and Phoridae) are still not entirely resolved. These other families are small, with few species known worldwide, in contrast to the hyperdiverse Phoridae. Most of the literature has considered the Ironomyiidae as their closest relatives^3–8^, although Lonchopteridae were more recently proposed to be closer to phorids within the Platypezoidea^9^.

**Figure 1:**
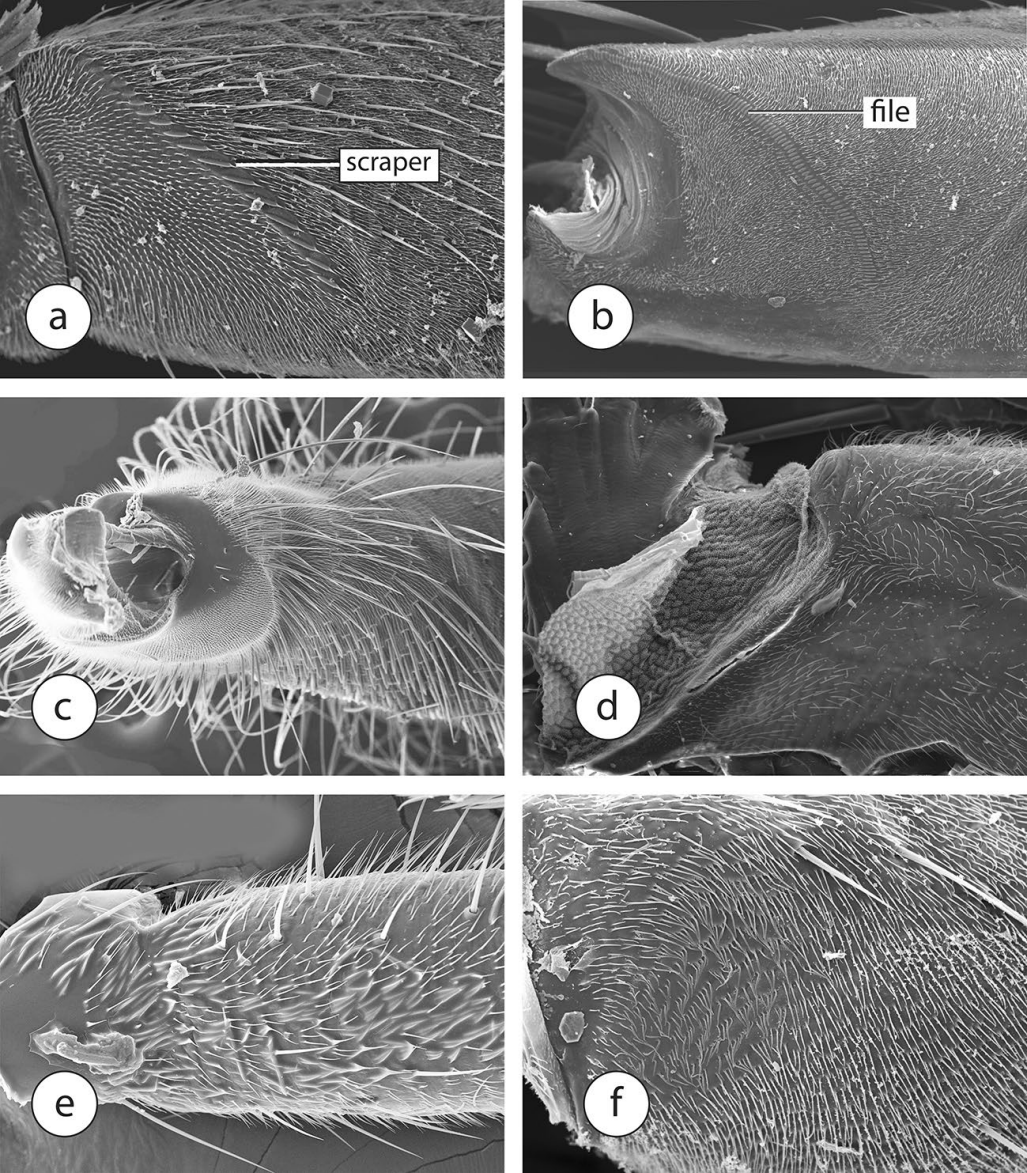
Scanning electron micrographs of forelegs of Diptera. (**a**, **b**) *Sciadocera rufomaculata*. (**a**) Forefemur, (**b**) forecoxa. (**c**–**f**) forefemora of Dolichopodidae, (**d**) *Rhamphomyia* sp., (**e**) *Brachystoma* sp., (**f**) *Ironomyia francisi*.

Grimaldi^10^ discovered a new character in the foreleg of sciadocerine phorids, which he referred to as a stridulatum, and hypothesized that it was used in the production of sound. He found it to be present in both adult males and females of extant sciadocerines, as well as in some related early fossil phorid species. This structure consists of a row of scalelike sculpturing on the inner face of the base of the forefemur (called the “scraper” by Grimaldi) that apparently are rubbed against a carinate ridge (the “file” of Grimaldi) on the outer face of the apex of the forecoxa (Fig. 1a,b). Based on Grimaldi’s discovery we checked specimens of non-sciadocerine phorid representatives in the collection of the Natural History Museum of Los Angeles County (LACM; Table 1) and we also examined a number of outgroups to check how widespread these structures could be in the platypezoids or other cyclorrhaphan flies.

**Table 1:** Material prepared and examined with SEM in this study.

Family	Genus or species	Country
Ironomyiidae	*Ironomyia francisi*	Australia
Lonchopteridae	*Lonchoptera* sp.	Slovakia
Platypezidae	*Microsania* sp.	USA
	*Lindneromyia* sp.	Chile
Opetiidae	*Opetia nigra*	Netherlands
Phoridae	*Sciadocera rufomaculata*	New Zealand
	*Gymnophora spiracularis*	Costa Rica
	Termitoxeniinae sp.	Malawi
	*Chonocephalus* sp.	Costa Rica
	*Latiborophaga* sp.	Costa Rica
	*Cremersia* sp.	Costa Rica
Dolichopodidae	unidentified species	USA
Empididae	*Rhamphomyia* sp.	USA
	*Brachystoma* sp.	USA
Syrphidae	unidentified species	USA
Tachinidae	unidentified species	USA

## Materials and methods

Specimens were collected by Malaise traps and chemically dried using hexamethyldisilazane^11^. They were sputter-coated with gold/palladium using an Emitech K550X and examined with a JEOL IT200LA scanning electron microscope.

## Results

Both components of the stridulatum are present in *Opetia* (Opetiidae) but missing in all non-phorid outgroups. In other Eremoneura (Empidoidea + Cyclorrhapha), there are often groups of modified microtrichia (Fig. 1c–e), but not modified into the scalelike structures in the stridulatum of phorids and opetiids.

**Figure 2:**
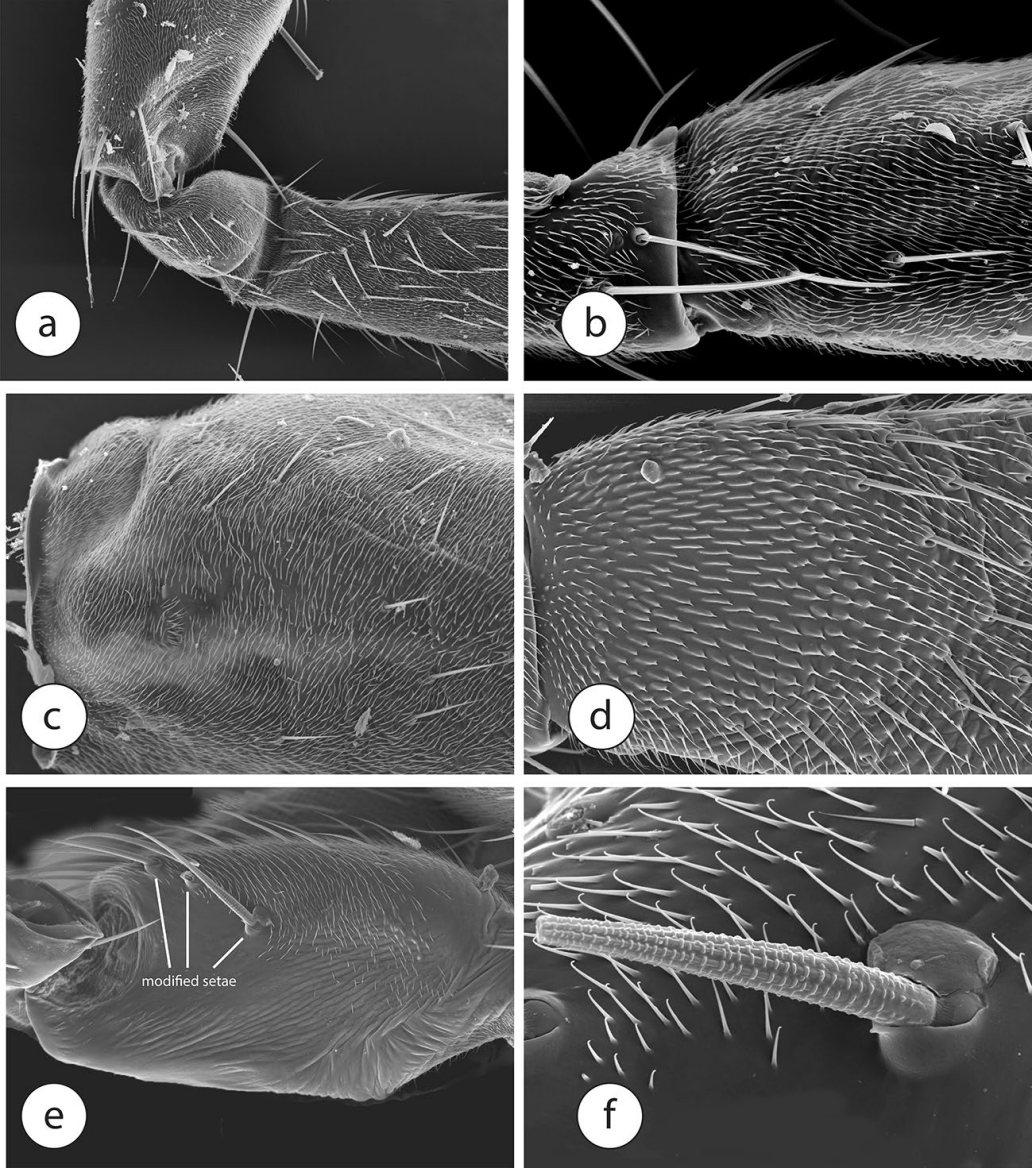
Scanning electron micrographs of forelegs of Diptera. (**a**–**d**) Forefemora, (**a**) *Lonchoptera* sp., (**b**) *Microsania* sp., (**c**) *Lindneromyia* sp., (**d**) *Opetia nigra*. (**e**, **f**) forecoxa *Opetia nigra*.

The usually implicated sister-taxon of the Phoridae, *Ironomyia* (represented in our data by *Ironomyia francisi* McAlpine) has no development of any stridulatum (Fig. 1f). There is some consolidation of microtrichia into groups of two to three, but this is not well developed. The lonchopterids studied by us did not vary significantly in structure (Fig. 2a) from other platypezoid taxa examined in the sense that there is no evidence of modification of the usual microtrichia towards the development of a stridulatum. Platypezids likewise have plain forefemora (Fig. 2b,c).

Specimens of *Opetia nigra* have a plausible precursor to the forefemoral scraper, formed by flattened microtrichia distributed across the inner surface of the forefemur (Fig. 2d). The forecoxa has no file, however, and such a structure also is not found in other lower cyclorrhaphan flies. Instead, *Opetia nigra* has three large setae of the forecoxa at that position, which are highly modified, with extremely unusual transverse ridges (Fig. 2e,f) and a peculiar socket structure. Unlike the sockets of most setae, which are circular and allow movement in every direction, these sockets are expanded on two sides, like a doorstopper. This apparently makes the setae more stable and effective when rubbed by the scales of the forefemur. It is possible that the integration of these transversely ridged setae into the surface of the forecoxa was through the continued incorporation of the setae through the fusion of the sockets.

**Figure 3:**
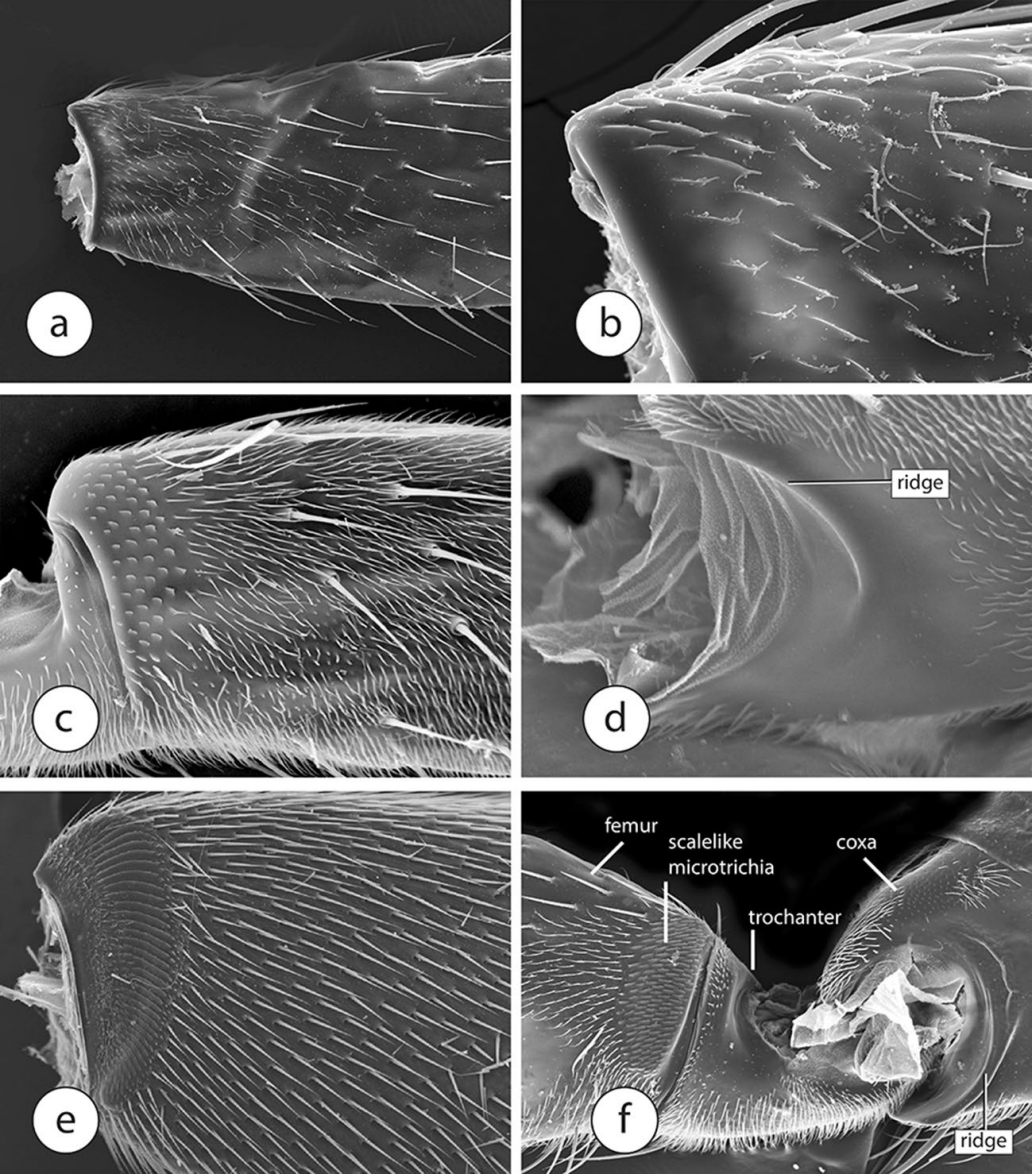
Scanning electron micrographs of forelegs of Phoridae. (**a**, **b**) forefemur of Termitoxeniinae, (**c**, **d**) *Chonocephalus* sp., (**c**) forefemur., (**d**) apex of forecoxa, (**e**) *Latiborophaga* sp., (**f**) foreleg of *Gymnophora* sp.

Within the Sciaodocerinae, the stridulatum consists of a distinct, curved line of scalelike microtrichia on the forefemur and an even more distinct, curved, ribbed line on the forecoxa. In the phorid subfamily Termitoxeniinae, the stridulatum is less developed than that in *Opetia*, with the scalelike microtrichia only slightly differentiated from other surrounding microtrichia (Fig. 3a,b), and without the file. Phorid flies of other subfamilies have a more modified stridulatum, derived from a condition like that seen in termitoxeniines. The Chonocephalinae, another early diverging branch of phorids, also have small, scalelike microtrichia (Fig. 3c) on the forefemur, but do not have a transversely ribbed file, either.

**Figure 4:**
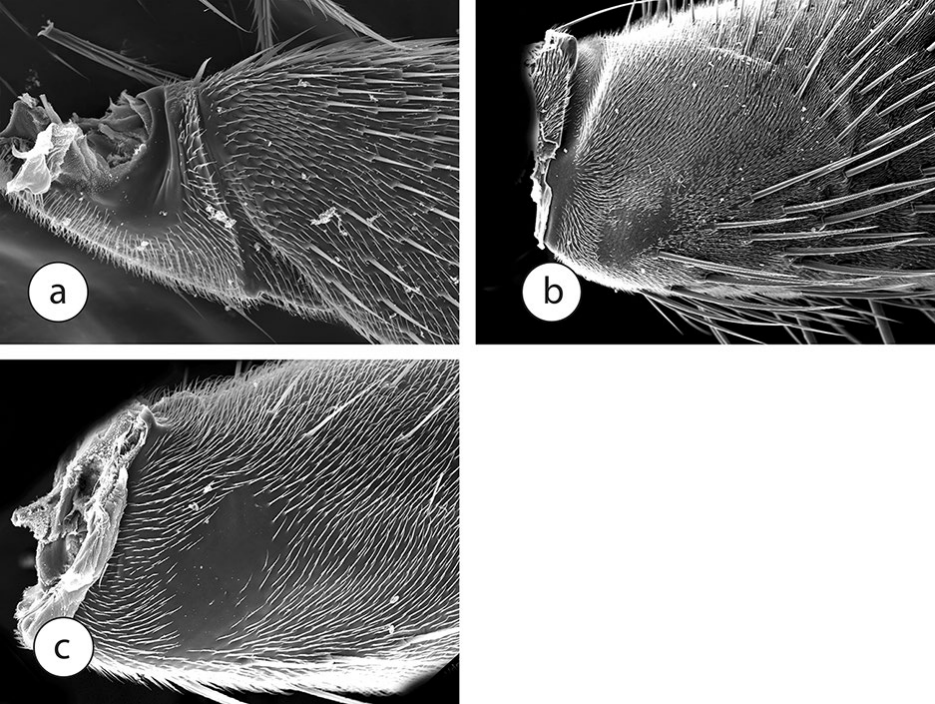
Scanning electron micrographs of forefemora of Diptera. (**a**) *Cremersia* (Phoridae), (**b**) Tachinidae, (**c**) Syrphidae.

We have two hypotheses about the origin of sciadocerine file. One is that it is a newly-derived structure, formed from differentiated microtrichia. The second is that the ribbed setae of opetiids are fused into the structure of the forecoxa through continued expansion of the setal base. This would be a mechanism for making the forecoxal rigidity even greater. It would be an almost unbelievable coincidence if the ribs of the file (a unique structure) are not derived from the ribbed setae (another unique structure) in the ancestor of Opetiidae and Sciadocerinae. Regardless, the function of being the surface against which scales are rubbed is taken over by the smoother ridge in its place (Fig. 3d) on the forecoxae of all other non-sciadocerine phorids examined. The largest scalelike microtrichia are found in the hypocerine phorids (subfamily Phorinae), in which these structures extend over a significant portion of the forefemur (Fig. 3e). In the largest phorid subfamily, the Metopininae, the scalelike microtrichia are varied in structure. They are usually small and often rounded (Fig. 3f), but in some genera they do not differ greatly from the corresponding structure seen in opetiids (Fig. 4a). In the metopinine genus *Cremersia*, for example, the scales are much larger and broader, with long pointed apices that are directed distally on the forefemur. Given this similarity between *Cremersia* and *Opetia*, with the scalelike microtrichia retaining long seta-like apices, and the condition seen in phorines, it seems that the smaller microtrichia in termitoxeniines are the result of secondary reduction. The variation of the morphological pattern of the stridulatum seen in different groups of phorids suggests that the sound produced by this structure also varies in different genera.

Other cyclorrhaphan families include species showing modifications of the forefemur, but none like in Opetiidae and Phoridae. Usually, the microtrichia are aligned in a pattern, often in pairs or triplets, or otherwise differentiated (Fig. 4b,c).

## Conclusions

These specific structures of the forecoxa and the forefemur appear nowhere else in the platypezoid families (or in other flies) and provide evidence supporting the hypothesis that these setae on the forecoxa of *Opetia* are modified within the stem phorids to form the file, seen in extant sciadocerines and in *Sciadocera*-like Mesozoic genera of phorids. In particular, the setae of the forecoxa in *Opetia* are highly unusual, with transverse sculpturing. Longitudinal sculpturing and feathering, in contrast, are found in setae of many phorids and are useful indicators of homology and relationships^9,12^. This file in sciadocerines is much more rigid than that of *Opetia* and allows more precision of the interaction of the stridulatum components. It is remarkable that the specific structure of the sciadocerines was later lost by some phorid clades, in which the ridge is not transversely ribbed, although this may have allowed the development of a larger field of forefemoral scalelike microtrichia, in contrast to the single line found in sciadocerines.

Although this region of the foreleg is not as highly modified in other Diptera, it does not preclude other groups of flies from using the structures found there, often differentiated microtrichia, as a stridulatory organ. Paired or otherwise organized microtrichia form grooves or patterns in the same position in other flies (Fig. 4b,c). Stridulatory structures are also known in other groups of flies, e.g., Agromyzidae, but originated much later, in the Cenozoic^13,14^. The scalelike structure of the opetiid/phorid forefemur, however, is the most distinctive within the order.

## Data Availability

All data generated or analyzed during this study are included in this published article.
